# Impact of exacerbations of cystic fibrosis on muscle strength

**DOI:** 10.1186/1465-9921-14-46

**Published:** 2013-04-19

**Authors:** Chris Burtin, Hans Van Remoortel, Bart Vrijsen, Daniel Langer, Kristine Colpaert, Rik Gosselink, Marc Decramer, Lieven Dupont, Thierry Troosters

**Affiliations:** 1 Faculty of Kinesiology and Rehabilitation Sciences, Katholieke Universiteit Leuven, Leuven, Belgium; 2 Respiratory Rehabilitation and Respiratory Division, University Hospitals KU Leuven, Herestraat 49 bus 706, Leuven, B3000, Belgium; 3 Adult Cystic Fibrosis Centre, University Hospitals KULeuven, Leuven, Belgium

**Keywords:** Muscle function, Physical activity, Cystic fibrosis, Exacerbation, Hospitalization, Magnetic stimulation, Quadriceps strength

## Abstract

**Background:**

Adult patients with cystic fibrosis have peripheral muscle weakness, which is related to exercise intolerance and poor prognosis. The influence of acute exacerbations on muscle strength has been poorly studied. This study aimed to investigate whether quadriceps force (QF), as assessed with an involuntary technique, changes during intravenous antibiotics therapy (IVAT) for an exacerbation.

**Methods:**

QF was measured in 20 patients using twitch stimulation of the femoral nerve at the day of hospitalization (day 1) and at termination (day 14) of the IVAT. Physical activity was monitored during IVAT using a SenseWear armband. Ten stable patients served as control subjects.

**Results:**

QF did not change during exacerbation (potentiated twitch force at day 1: 140 ± 42 N, at day 14: 140 ± 47 N), but a decrease was observed in individual patients. Changes in twitch force during exacerbation were correlated with time spent in activities of at least moderate intensity (r = 0.61, p = 0.007).

**Conclusions:**

QF does not systematically decrease during exacerbations of cystic fibrosis. Individual changes in QF are well correlated with daily time spent in activities of at least moderate intensity.

## Background

Peripheral muscle weakness is prevalent in adult patients with cystic fibrosis (CF) [1-3]. A 25 to 35% decrease in quadriceps strength is observed in comparison with age-matched healthy adults [1-3]. Quadriceps weakness is directly related to exercise intolerance in these patients [3]. Furthermore decreased muscle mass and exercise intolerance are associated with poor prognosis [4-6]. Physical inactivity is likely to be an important underlying factor of the observed muscle weakness [3].

CF is characterized by the occurrence of pulmonary exacerbations which are associated with increased respiratory symptoms. Frequent exacerbations negatively influence quality of life [7], prognosis [8] and accelerate lung function decline [8,9]. Exacerbations can potentially aggravate factors underlying muscle dysfunction including physical inactivity, systemic inflammation and anabolic status [10,11]. Wieboldt et al. recently reported that quadriceps strength at hospital admission was eight percent lower than at convalescence one month after hospital discharge in CF patients admitted for an acute exacerbation [12]. No relationship was observed between physical activity levels during hospitalization and measures of muscle strength. Muscle strength was lower at hospital admission compared to discharge, whereas one would expect a decrease in muscle strength during the hospitalization due to observed inactivity during an acute exacerbation, as has been observed in COPD [13]. In the study of Wieboldt et al., quadriceps strength was measured by means of maximal voluntary contractions [12]. As patients experience an increase in symptoms during an exacerbation [14], the performance during a maximal volitional test at the day of hospital admission might be attenuated. Consequently, an involuntary test is perhaps a more appropriate approach to quantify changes in muscle strength during hospitalization for an exacerbation. Magnetic stimulation of the femoral nerve is a valid technique to provide an involuntary measurement of quadriceps force [15]. In addition, it would be worthwhile to objectively document physical activity levels in terms of their intensity during and after the acute exacerbation.

Therefore, this prospective case–control study investigates whether peripheral muscle strength, assessed with an involuntary technique, is attenuated by an acute exacerbation and whether changes in muscle strength during exacerbation can be linked with physical activity levels. We hypothesized that quadriceps force would decrease in inactive patients.

## Methods

### Subjects

A convenience sample of 20 adult patients with CF that were admitted to the University Hospital Gasthuisberg in Leuven for an acute exacerbation were recruited. An acute exacerbation was diagnosed based on the criteria of Fuchs et al. [16]. Patients with four or more of the following signs or symptoms were considered to have an exacerbation and were treated with intravenous antibiotics: 1) increased cough; 2) increased dyspnea; 3) change in sputum; 4) new/increased hemoptysis; 5) temperature > 38°Celsius; 6) anorexia or weight loss; 7) malaise/fatigue/lethargy; 8) radiographic changes indicative of pulmonary infection; 9) change in physical examination of the chest; 10) > 10% decline in pulmonary function from previous measurement; 11) sinus pain/tenderness; 12) change in sinus discharge. All patients received intra-venous antibiotic therapy (IVAT) for 14 days. A hospitalization period of 14 days was foreseen.

Ten stable CF patients without acute exacerbation in the preceding six months were included as stable control patients. These patients were recruited during their annual follow-up visit in the hospital and were matched with the exacerbation group for age and gender.

Exclusion criteria were the presence of orthopedic conditions interfering with the assessment of skeletal muscle force, the occurrence of a life-threatening exacerbation, an operation in the inguinal region in the previous two months (contra-indication to perform magnetic stimulation) and inclusion in a structured exercise training program.

Written informed consent was obtained from all patients. The study protocol was approved by the local ethics committee of University Hospital Gasthuisberg, Leuven.

### Study design

Muscle strength assessment, spirometry and venous blood sampling were performed at the beginning (day 1) and at the end of IVAT (day 14) and one month after IVAT (day 40 ± 3 days). Six-minute walking distance (6MWD) was assessed at day 14 and day 40. Physical activity levels were measured using activity monitoring during (from day 1 till day 14) and one month after the exacerbation (two weeks starting from day 40).

The control patients performed the same testing procedures (muscle strength, spirometry, 6MWD) at the time of recruitment and 14 days later. Physical activity was recorded for two weeks between the two measurement sessions.

### Assessment of quadriceps strength

The quadriceps force was evaluated using maximal voluntary contraction and transcutaneous magnetic twitch stimulation of the right femoral nerve. Subjects were sitting in a recumbent chair with hips extended at 120°, knees flexed at 90° and arms crossed in front of the chest. The following measures were performed in a fixed order to obtain a comprehensive assessment of skeletal muscle force (i.e. voluntary and involuntary muscle strength measurement):

Unpotentiated Quadriceps Twitch contraction

At rest, the right femoral nerve was stimulated through a 45 mm figure-of-eight coil powered by a double Magstim stimulator (Magstim Co Ltd., Whitland, Dyed, Wales, UK). The strain-gauge signal was transformed by an analogue force transducer (DS Europe 546QD), amplified (Model 811A amplifiers; Hewlett-Packard) and stored on a computer. Twitch forces were measured at 30, 50, 70, 80, 90, 95 and 100% of the maximum stimulator output and supramaximality was shown by reaching a plateau in force output. Supramaximality was achieved in all patients.

Maximal Voluntary Contraction (MVC)

Subjects performed 5 isometric MVCs for three seconds, with a resting period of at least thirty seconds between trials For analysis, the mean of the two highest peak force values was calculated.

Potentiated Quadriceps Twitch contraction

The potentiated quadriceps twitch force (TWq_pot_) was systematically measured three seconds after the end of each MVC maneuver. The femoral nerve was stimulated with a twitch at 100% of power output of the stimulator. For analysis, the mean of the two highest values was calculated.

### Assessment of physical activity

The SenseWear Pro Armband (BodyMedia, Inc., Pittsburgh, PA) was worn to quantify physical activity. The device (85×54×20 mm, 85 g) is placed on the upper right arm and integrates information from a biaxial accelerometer with signals from non-invasive sensors measuring physical parameters such as changes in body temperature, near body ambient temperature, heat flux, and galvanic skin resistance. Together with individual characteristics including gender, age, height and body mass these variables are used to estimate energy expenditure (expressed as metabolic equivalents of tasks, METs) utilizing proprietary equations developed by the manufacturer. After analysis of raw data the number of daily steps as well as daily time spent performing activities at various intensities can be evaluated. The time spent with an energy expenditure of >4.8 METS was considered as at least moderate intense physical activity. This cut-off was based on an age-based classification of physical activity intensity, as suggested by the US Department of Health and Human Services [17].

This device provides accurate estimates of daily number of steps and energy expenditure in patients with CF [18].

### Spirometry and six-minute walking distance

Dynamic lung volumes were measured according to the European Respiratory Society guidelines [19]. The 6MWD tests were performed in a 50m corridor with standardized encouragement [20].

### Blood analyses

Venous blood serum samples were analyzed for hemoglobin, blood platelets, testosterone, C-reactive protein and white blood cells (total count and neutrophils) content. Testosterone levels were not available in the stable patients.

### Statistical analysis

All statistical analyses were performed with SAS 9.2. Data were expressed as means ± standard deviations or medians [interquartile range] as appropriate. Moderate intense physical activity, testosterone levels and C-reactive protein were not normally distributed. The level of significance was 0.05 for all statistical tests. Repeated measures were analyzed using repeated-measures ANOVA (with Tukey post hoc tests), paired t-tests or Wilcoxon signed rank tests. Comparisons between independent groups of patients were made with unpaired t-tests or Mann–Whitney U tests where appropriate. Pearson or Spearman rank correlation coefficients were used to evaluate relationships between variables. Analysis of covariance was used to correct these relationships for baseline muscle strength.

## Results

One hospitalized patient did not comply with study procedures and was excluded from analysis, leaving 19 patients (25 ± 6 years, 37% female and body mass index (BMI) 20.9 ± 2.9) that were tested at day 1 and day 14. IVAT was administered for the full 14 days in the hospital in eleven patients, while eight patients received IVAT at home from day 4 onwards. Seventeen patients were tested at day 40, as two patients refused to attend for the follow-up visit. One patient had a nickel allergy which made it impossible to wear the activity monitor.

The ten stable CF patients (controls) had a mean age of 29 ± 8 years, 40% were female and BMI was 20.4 ± 2.0. One stable patient did not comply with the physical activity measurements.

Baseline characteristics of hospitalized and stable patients are shown in Table 1, No statistical differences were found, but chronic Pseudomonas Aeroginosa colonization tended to be less frequent and CF-related diabetes tended to be more frequent in the exacerbation group.

**Table 1:** Baseline characteristics

	**Patients with exacerbation**	**Stable patients**	**p-value**
	**(n = 19)**	**(n = 10)**	
Age (yrs)	25 ± 6	29 ± 8	0.14
Gender (male/female)	13/6	6/4	0.70
BMI (kg/m^2^)	20.9 ± 2.9	20.4 ± 2.0	0.63
Chronic PA colonization (n)	14 (74%)	10 (100%)	0.07
CF-related diabetes mellitus (n)	11 (58%)	2 (20%)	0.051
Pancreas insufficiency (n)	16 (84%)	9 (90%)	0.67
Genotype ΔF508/ΔF508 (n)	12 (63%)	5 (50%)	0.49
# Exacerbations in 12 months prior to inclusion	1 [0–2]	0 [0–1]	0.09

Data are presented as mean ± standard deviation or median [interquartile range]. BMI = body mass index, PA = Pseudomonas Aeruginosa, CF = cystic fibrosis.

The results regarding spirometry, quadriceps strength assessment, 6MWD, daily physical activity hematological parameters, testosterone levels and systemic inflammation at different time points during and after the exacerbation and in stable patients are summarized in Table 2. Forced expiratory volume in one second (FEV_1_) recovered during the exacerbation (p < 0.001) to reach the level of stable patients at day 14. At day 40, FEV_1_ was decreased again to some extent (p < 0.05).

**Table 2:** Spirometry, quadriceps strength, functional exercise capacity, physical activity levels and blood serum parameters in patients during a respiratory exacerbation and in stable patients

			**Exacerbation**	
	**During exacerbation**		**Follow-up**	**Stable**
	**(n = 19)**		**(n = 17)**	**(n = 10)**
	**Day 1**	**Day 14**	**Day 40**	
**Spirometry**				
FEV_1_ (L)	2.1 ± 0.9**^,#^	2.7 ± 1.1^#^	2.4 ± 1.0	2.8 ± 1.0
FEV_1_ (%pred)	55 ± 19**^,##^	69 ± 25^#^	61 ± 23	72 ± 22
FVC (L)	3.5 ± 1.0**	4.0 ± 1.2	3.7 ± 1.2	4.1 ± 0.8
**Quadriceps strength and functional exercise capacity**				
Twq_pot_ (N)	140 ± 42	140 ± 47	129 ± 35	158 ± 40
MVC (N)	378 ± 128	369 ± 119	353 ± 109	456 ± 157
6MWD (m)	/	694 ± 89^§^	698 ± 84^§^	786 ± 111
**Physical activity**				
Daily steps (n)	4654 ± 2929^#,§^	7174 ± 4423	7414 ± 2810	
Time > 4.8 METs (min)	6 [2-17] ^#^	14 [4–40]	11 [6–35]	
**Hematological parameters, testosterone and systemic inflammation**				
Hemoglobin (g/dL)	13.9 ± 1.5	13.7 ± 1.6	13.8 ± 1.4	14.8 ± 1.2
Blood platelets (x10^9^/L)	314 ± 109*	287 ± 104	339 ± 87	250 ± 63
Testosterone (in men) (ng/dL)	249 [148–281]	354 [251–442]	256 [209–363]	/
WBC count (x10^9^/L)	12.6 ± 4.3**^,#,§^	8.6 ± 3.1	9.9 ± 2.3^§^	7.4 ± 2.2
Neutrophil count (x10^9^/L)	8.6 ± 3.0**^,§^	5.2 ± 2.4	7.1 ± 2.4^§^	4.7 ± 1.8
CRP (mg/L)	13 [8–48]**^,§^	3 [0–5]	9 [7-18] ^§^	2 [0–7]

Data are presented as mean ± standard deviation or median [interquartile range].

Data in the stable group are data from the first assessment, except for physical activity measurements, which are the mean values over a 14-day assessment period.FEV_1_ = forced expiratory volume in one second; %pred = percentage of the predicted value; FVC = forced vital capacity; Twq_pot_ = potentiated quadriceps twitch force; MVC = maximal voluntary contraction force of the quadriceps, 6MWD = six-minute walking distance, MET = metabolic equivalent, WBC = white blood cells, CRP = C-reactive protein. *p < 0.05 vs. day 14 **, p < 0.001 vs. day 14, ^#^ p < 0.05 vs. day 40, ^##^ p < 0.001 vs. day 40, ^§^p < 0.05 vs. stable.

### Quadriceps strength

**Figure 1:**
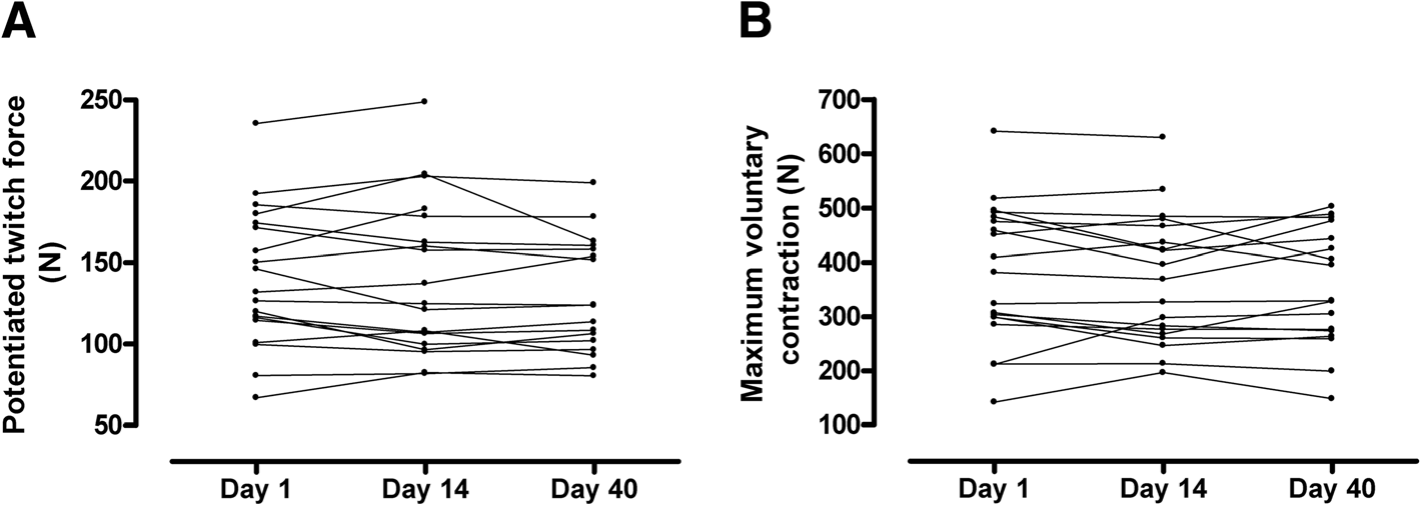
Individual quadriceps strength, represented as potentiated twitch force (1A) and maximum voluntary contraction (1B), during exacerbation and one month after exacerbation (day 40).

Quadriceps strength did not change significantly during exacerbation or follow-up (Figure 1A and B) and was comparable with strength observed in stable patients.

At day 40, potentiated twitch force tended to be lower in the exacerbation group compared with stable patients (129 ± 35 vs. 158 ± 40, p = 0.06). Observations on baseline values and changes in muscle strength were similar between patients receiving IVAT in the hospital or partly at home.

At day 1, potentiated twitch force and MVC were significantly related to testosterone levels at day 1 (both r = 0.82, p < 0.0001), but not with inflammatory markers (white blood cells, neutrophils, C-reactive protein).

**Figure 2:**
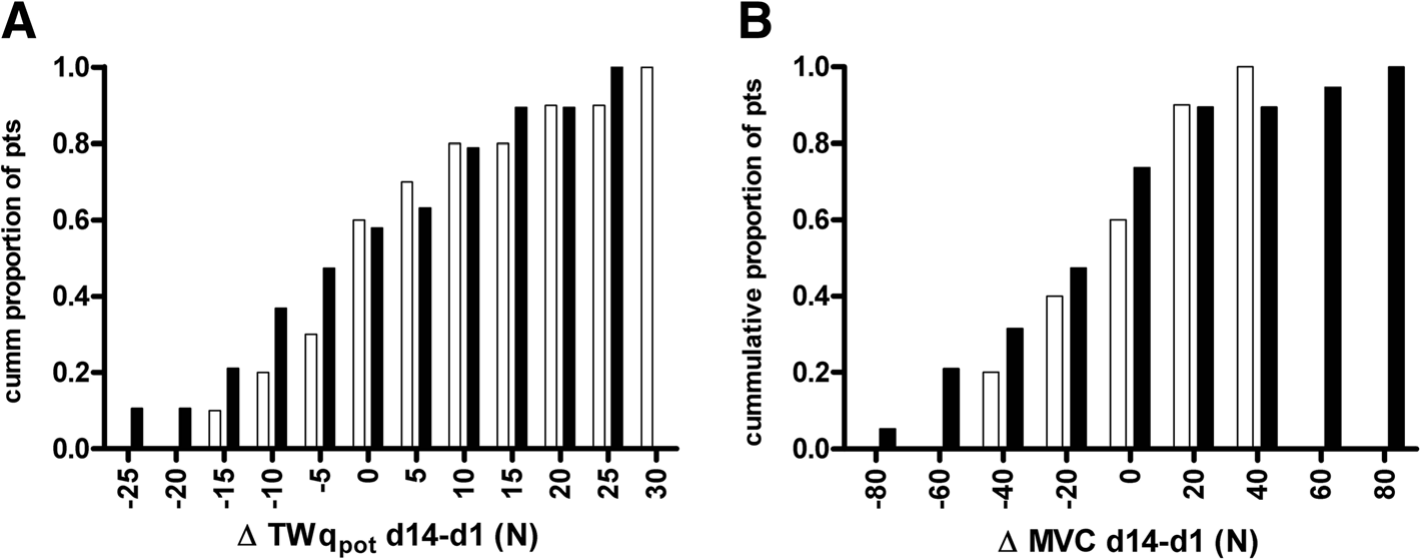
Cumulative histogram of changes in potentiated twitch force (TWqpot, 2A) and maximum voluntary contraction (MVC, 2B) between day 1 and day 14 in patients during exacerbation (black bars) and stable patients (white bars).

The absolute value of the change in muscle force between day 1 and day 14 in patients with exacerbations (Twq_pot_ 7 [5-13]%; MVC 6 [0–15]%) was comparable with the random variability observed with the two strength measurements in the control group (Twq_pot_ 6 [2-10]%; MVC 6 [3-8]%). Figure 2 shows the distribution of the change in twitch force (Figure 2A) and MVC (Figure 2B). In the exacerbation group a numerically higher proportion of patients had a larger decrease in muscle strength over the 14 days.

### Daily physical activity

**Figure 3:**
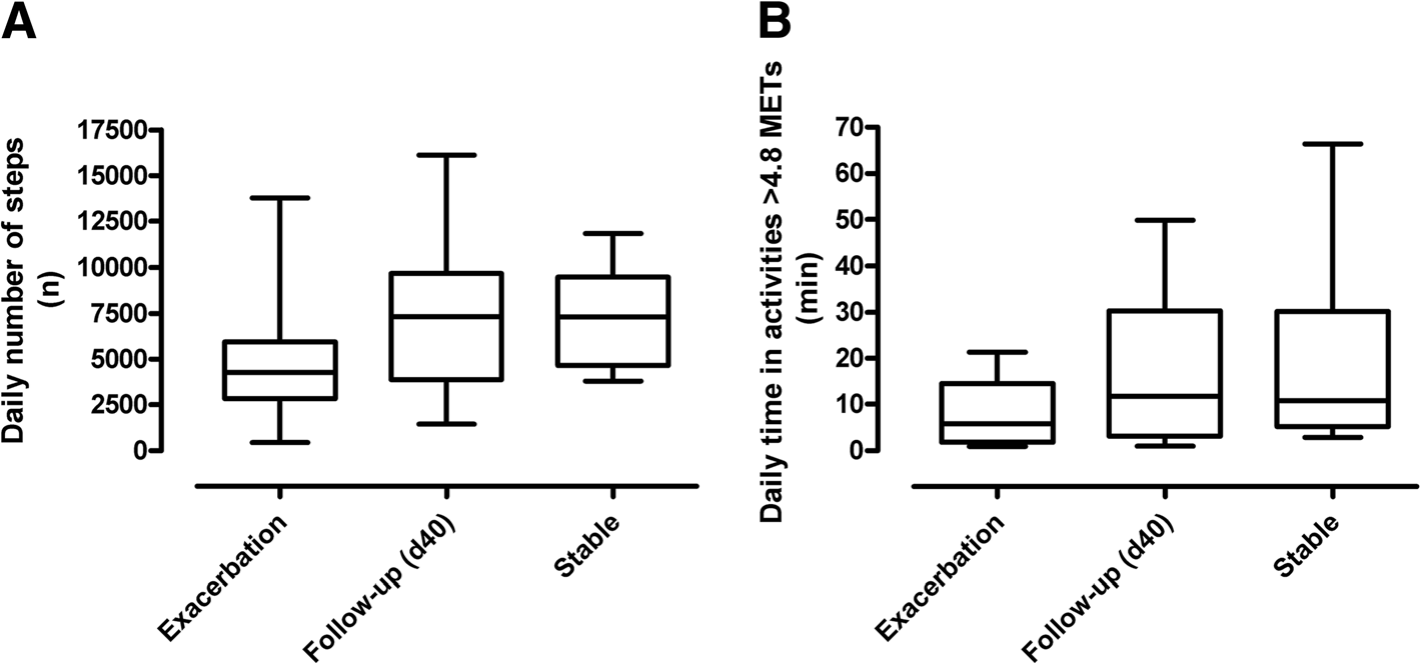
**Individual physical activity levels (time spent in activities with an energy demand > 4.8 MEts indicating moderate intense physical 3A; daily number of steps, 3B) during and one month after an exacerbation and in stable patients.** *p<0.05 vs. day 40, # p<0.05 vs stable patients.

The amount of daily steps and daily time spent in at least moderate intense activities was lower during the exacerbation but increased to the level of stable patients during follow-up (p < 0.05, Figure 3A and B). Physical activity levels were not significantly different between patients receiving IVAT in the hospital (4283 [3276–5629] steps/day and 6 [2-17] min spent in activities above 4.8 METs) and patients partly receiving IVAT at home (3517 [2411–7468] steps/day and 4 [2-18] min spent in activities above 4.8 METs). No difference in activity levels was observed between males and females.

### Systemic inflammation and testosterone

White blood cell, neutrophil and CRP concentrations were elevated at day 1 and normalized by day 14. At day 40, these parameters tended to increase again and white blood cell and neutrophil count were significantly higher compared with stable patients (p < 0.05). Serum testosterone levels in men with CF remained unchanged during the exacerbation.

### Changes in quadriceps strength during IVAT, physical activity and systemic inflammation

**Figure 4:**
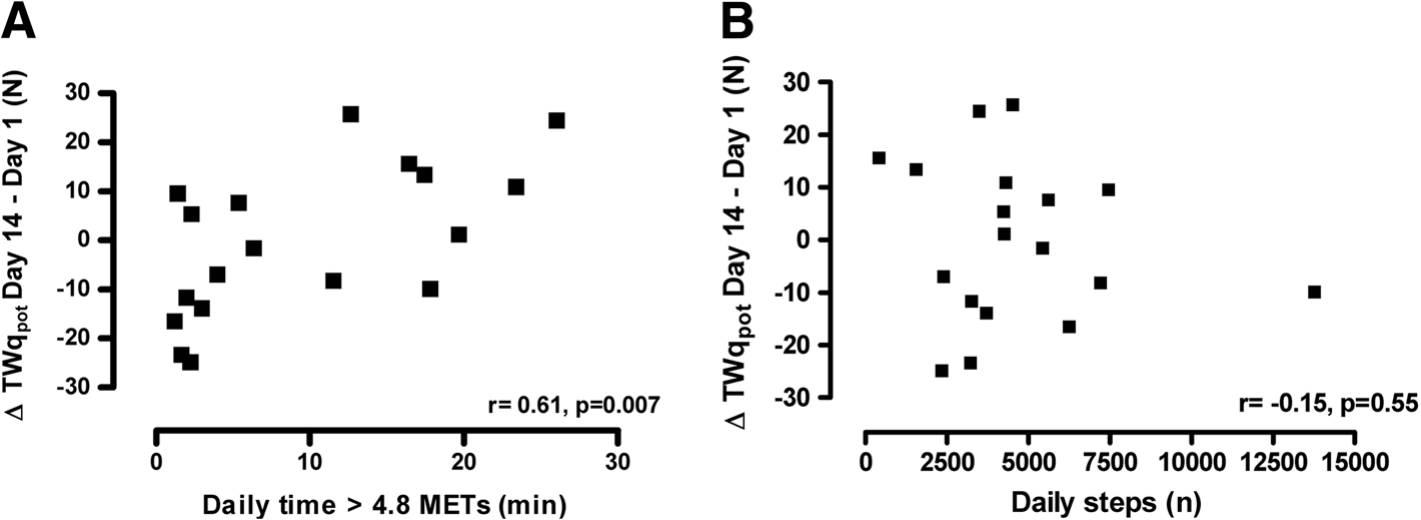
Relationship between daily physical activity (time spent in activities with an energy demand > 4.8 METs indicating moderate intense physical activity, 4A; daily number of steps, 4B) and change in potentiated twitch force (TWqpot) during an exacerbation.

Change in TWq_pot_ was significantly related with moderate intense physical activity during exacerbation (r = 0.61, p = 0.007, Figure 4A), but not with the daily number of steps (r = −0.15, p = 0.55, Figure 4B). Seventy percent of patients who spent less than 10 minutes per day in moderate intense physical activity showed a decrease in twitch force, compared to 25% of patients with more daily moderate intense activity (p = 0.057). Change in MVC was not correlated with measures of physical activity during exacerbation (r = 0.29, p = 0.24 for moderate intense physical activity; r = −0.09, p = 0.72 for daily steps). In the stable patients, the difference in muscle force between the two measurements was not related with physical activity or blood parameters after correction for baseline muscle strength.

No relationships were observed between hematological variables, testosterone levels and parameters of systemic inflammation on the one hand and changes in muscle strength on the other hand.

## Discussion

This study shows that quadriceps force does not systematically decrease during exacerbations of CF. Interestingly, the daily time spent in at least moderate intense activities is closely linked with individual changes in quadriceps twitch force during the same period.

### Muscle strength

Quadriceps weakness is known to be present in more than 50% of stable patients [3], but the contribution of exacerbations to the development of such weakness is unknown. We observed no systematic detrimental impact of an acute exacerbation on muscle force. A widespread variability of quadriceps force change is observed among CF patients. This observation is not in line with a recent study where quadriceps strength, as measured by volitional maximal maneuvers, was significantly increased in adult CF patients during hospital admission for an acute exacerbation (14). By definition, patients with CFexperience an increase in symptoms at the onset of an exacerbation [21]. A recent study shows that 93% of patients reported increased dyspnea and 84% reported feelings of malaise during acute exacerbations [14]. These symptoms potentially have an influence on the result of a maximal volitional test of muscle strength, especially at hospital admission. This could possibly explain the observed increase in muscle strength during exacerbation in the study of Wieboldt et al. [12]. Interestingly, our results did not confirm the findings of Wieboldt et al., as no systematic change in MVC was observed. Anyhow, the use of magnetic stimulation of the femoral nerve to assess quadriceps strength has the potential to overcome the possible influence of increased symptoms on muscle strength measurements. This technique is an involuntary, well-tolerated and valid technique to assess quadriceps strength in healthy subjects and patients with chronic obstructive pulmonary disease [15,22,23].

The variability of the change in muscle force in CF patients during an exacerbation was similar to the random between-measurement variability found in stable CF patients, although in the exacerbation group more patients appeared to have a pronounced decrease in muscle force. Similar variability of quadriceps force assessments (both maximal voluntary maneuvers and magnetic twitch force) has been reported [23,24].

### Physical activity

During IVAT, both the amount (daily steps) and the intensity (time spent in activities of at least moderate intensity) of daily physical activity are reduced compared to stable controls. The daily number of steps was 36% lower in patients with an exacerbation compared with stable controls, whereas time spent in activities of at least moderate intensity was even more reduced. Hospitalization itself might be a reason underlying this marked inactivity, as patients spent their days indoor. Interestingly, however, patients that continued IVAT at home from day 4 onwards were not more active than patients that completed IVAT in the hospital. The IVAT and the increased respiratory symptoms are possible reasons for the observed inactivity. One month after the exacerbation, physical activity increased to the level of stable patients, but especially time spent in at least moderate intense activities was still clearly below levels observed in healthy age-matched adults in literature (median 14 min/day vs. 35 min/day) [3].

Whereas individual changes in twitch force during antibiotic therapy were strongly correlated with daily time spent in activities of at least moderate intensity, no relationship was found with the daily number of steps. This is in line with previous observations of our group that time spent in moderate intense physical activity but not daily number of steps is related with exercise tolerance and quadriceps force in patients with stable CF (3). Moreover, the recent study of Wieboldt et al. demonstrated that a reduction in quadriceps strength during an acute CF exacerbation did not correlate with daily steps, however, the relationship with moderate intense physical activity was not investigated [12]. As showed in our study, patients with very low levels of moderate intense physical activity (less than ten minutes per day) seem more vulnerable to a decrease in muscle force.

### Systemic inflammation and testosterone

Although white blood cell, neutrophil and C-reactive protein serum concentrations are clearly increased at the onset of the exacerbation, no relationship is observed between blood parameters of inflammation and muscle force. Unfortunately we did not take muscle biopsies to investigate local muscle inflammation. Serum testosterone levels were strongly related to quadriceps strength, which is not in line with the findings of Barry et al. [25]. This might be explained by the observation that over 75% of our male patients had low testosterone levels (<300 ng/dL) whereas Barry et al. reported normal levels. However, testosterone levels did not change over time and were not related to changes in quadriceps force.

### Future research

It is tempting to speculate that moderate intense exercise training could be an interesting intervention to prevent deterioration of the muscle in those patients that do not voluntarily engage in these activities. This might be particularly important in patients with frequent exacerbations. The impact of repeated respiratory exacerbations on muscle function has not yet been investigated in patients with CF. If, due to respiratory symptoms, physical activity is difficult to achieve, resistance training or neuromuscular electrical stimulation could perhaps be considered, as both are associated with a limited ventilatory load. These approaches have been shown to improve muscle strength in patients with a pulmonary infection [26] and stable patients with several pulmonary impairment [27,28].

## Conclusions

Quadriceps strength does not systematically decrease during exacerbations of cystic fibrosis. Individual changes in quadriceps strength are well correlated with the daily time spent in activities of at least moderate intensity.

## Abbreviations

QF: Quadriceps force; IVAT: Intravenous antibiotic therapy; COPD: Chronic Obstructive Pulmonary Disease; CF: Cystic Fibrosis; 6MWD: Six-minute walking distance; MVC: Maximal voluntary contraction; TWqpot: Potentiated twitch force; METs: Metabolic equivalent of task; ANOVA: Analysis of variance; BMI: Body mass index; FEV1: Forced expiratory volume in 1 second; CRP: C-reactive protein.

## Competing interest

All authors declare not having any financial and personal relationships with other people or organisations that could inappropriately influence our work.

## Authors’ contributions

CB contributed to the protocol development, collected the data, performed data analysis and wrote the manuscript. HVR contributed to the protocol development, collected the data, performed data analysis and wrote the manuscript. BV contributed to the protocol development, assisted in the data collection and critically reviewed the manuscript. DL contributed to the protocol development, assisted in the data collection and critically reviewed the manuscript. KC assisted in the data collection and critically reviewed the manuscript. RG contributed to the protocol development and critically reviewed the manuscript. MD contributed to the protocol development and critically reviewed the manuscript. LD provided the study idea, contributed to the protocol development and critically reviewed the manuscript. TT provided the study idea, contributed to the protocol development and critically reviewed the manuscript. All authors read and approved the final manuscript.
